# Association between Serum Cortisol and DHEA-S Levels and Response to Antipsychotic Treatment in Schizophrenia

**DOI:** 10.3889/oamjms.2015.018

**Published:** 2015-02-22

**Authors:** Zoja Babinkostova, Branislav Stefanovski, Danijela Janicevic-Ivanovska, Valentina Samardziska

**Affiliations:** 1 *University Clinic of Psychiatry, Biological Psychiatry, Faculty of Medicine, Ss Cyril and Methodius University of Skopje, Skopje, Republic of Macedonia*; 2 *Institute of Clinical Biochemistry, Faculty of Medicine, Ss Cyril and Methodius University of Skopje, Skopje, Republic of Macedonia*

**Keywords:** schizophrenia, cortisol, DHEA-S, responders, nonresponders

## Abstract

**BACKGROUND:**

Previous studies suggested that alterations in serum cortisol and DHEA-S levels may play a role in the pathophysiology of schizophrenia.

**AIM:**

To compare serum cortisol and DHEA-S levels between patients with schizophrenia and healthy controls and to evaluate their association with the response to antipsychotic treatment.

**MATERIAL AND METHODS:**

In this clinical prospective study were included 60 patients with schizophrenia and 40 healthy age and sex matched control subjects. Clinical evaluation of patients was performed using the Positive and Negative Symptom Scale. A questionnaire for socio-demographic and clinical data collection was used. For the purposes of the study, the examined group was divided in two subgroups: responders and nonresponders. Serum cortisol and DHEA-S levels were measured at baseline in all participants and after 3 and 6 weeks of the antipsychotic treatment in patients with schizophrenia.

**RESULTS:**

Patients with schizophrenia had significantly higher serum cortisol and DHEA-S levels in comparison to the control group. Responders had significantly higher serum cortisol and DHEA-S levels compared with nonresponders.

**CONCLUSION:**

Elevated serum cortisol and DHEA-S levels may play a role in the pathophysiology of schizophrenia and they may be related to positive response to antipsychotic treatment in patients with schizophrenia.

## Introduction

The neuroendocrinologic system, particularly the HPA axis, has been a focus of interest for neurobiological studies aiming at elucidating the cause of schizophrenia [1]. HPA axis abnormalities may cause an increase in the baseline cortisol level [2]. It has been demonstrated that serum baseline cortisol levels are increased in patients with schizophrenia [1-10]. However there are also other studies with contrary findings [11-12].

Recently there has been increased interest in the role of dehydroepiadrosterone (DHEA) which, in its sulfated form (DHEA-S) is the most abundant in humans [13]. It is considered both a neurosteroid, being produced in the brain, as well as a neuroactive steroid, produced in the adrenals and gonads and having its effect on the brain [14]. Dehydroepiandrosterone sulfate (DHEA-S) is a neuroactive steroid interacting with N-methyl D-aspartate (NMDA) and gamma-aminobutyric acid (GABA) receptors [15]. Also DHEA-S may act as endogenous neuroprotective factor. The decline of its level during aging and schizophrenia may leave the brain unprotected against neurotoxic challenges [16]. Previous studies investigating DHEA-S blood levels in patients with schizophrenia have demonstrated elevated DHEA-S levels [2, 17-18], no different [11, 19] and decreased levels [20] in schizophrenia patients compared to healthy controls. The inconsistencies in published findings may be due to wide clinical polymorphism, small sample sizes or differences in the age and duration of illness of patients enrolled in the studies [21]. Authors of one study reported elevated serum DHEA-S levels in first episode male patients with schizophrenia compared to male patients with schizophrenia who were not in the first episode but were in a phase of acute exacerbation. According to their results they suggested that this neurosteroid response is unique to first-episode schizophrenia patients [22].

Previous studies have suggested that alterations in cortisol and DHEA-S levels may play a role in the pathophysiology of schizophrenia [2, 18, 23-26]. Serum cortisol and DHEA-S levels may be used as a biological marker for the diagnosis of schizophrenia; however, further studies with larger sample sizes are warranted to support this finding [2].

Authors of one study investigated association between serum cortisol, DHEA-S levels, as well as their molar ratios with the response to antipsychotic treatment in patients with schizophrenia during the exacerbation of the disorder [21]. They suggest that imbalance in serum cortisol and DHEA-S may be related to pathophysiological processes in schizophrenia, particularly to responsivity to antipsychotic treatment.

The aim of the study was to compare serum cortisol and dehydroepiandrosterone-sulfat levels between patients with schizophrenia and healthy control subjects and to evaluate association between these hormones and response to antipsychotic treatment in schizophrenic patients.

## Material and Methods

In this clinical prospective study by its design were included 60 patients with schizophrenia and 40 healthy age and sex matched control subjects.

Examined group consisted of sixty patients with schizophrenia from both genders; age 18-50, treated as inpatients or outpatients at the University Clinic of Psychiatry, Skopje, Macedonia. All patients experienced an acute exacerbation of the illness (PANSS: P1-Delusions and P3-Hallucinatory behavior ≥ 4). Patients who suffered from major physical illness, drug or alcohol abuse, epilepsy and other organic brain syndromes were not included. All patients underwent physical examination and routine laboratory tests to rule out physical illness. Clinical evaluation of patients was performed using the Positive and Negative Symptom Scale [27]. Non-standardized questionnaire was used for socio-demographic and clinical data collection.

For the purposes of this study, the examined group was divided in two subgroups:

1. subgroupof subjects suffering from schizophrenia classified as responders who had no ratings of >3 on items P1, P2, P3, P5 and P6 of the PANSS;
2. subgroup of subjects suffering from schizophrenia who did not meet these criteria were defined as nonresponders.

Control group consisted of forty healthy age and sex matched control subjects. All were physically healthy and had no personal or family history of psychiatric illness.

All participants in the study provided written informed consent to participate in this prospective study after having received a detailed explanation of the study procedures. The study was approved by the Ethics Committee of Medical University in Skopje and the Board of the University Clinic of Psychiatry.

### Steroid determination

Serum cortisol and DHEA-S levels were measured in the Institute of clinical biochemistry at the Medical University in Skopje. Serum samples of cortisol and DHEA-S were collected between 8 a.m. and 9 a.m. hours after 20 min of rest. All participants were instructed to abstain from unusual physical activity or stress for a period of 24 h prior to blood sampling. Blood samples were collected at baseline in all participants and after 3 and 6 weeks of the antipsychotic treatment in patients with schizophrenia. Cortisol and DHEA-S levels were measured by the IMMULITE 2000, competitive chemiluminescent enzime immunoassay.

### Statistical analysis

Several statistical methods have been used for the statistical analysis of the data obtained in the course of the study: non-parametric methods (Chi-square test, Mann-Whitney U test) and parametric methods (t-test for independent samples). From the multivariate methods MANOVA was used. Values of p < 0.05 were considered statistically significant.

## Results

Patients with schizophrenia had significantly higher mean serum cortisol and DHEA-S levels in comparison to the control group (Table 1).

**Table 1:** Serum levels of cortisol and DHEA-S in the examined and control group.

Hormone	Examined group	Control group	t-test	p-value
Cortisol (nmol/L)	555.7 ± 159.8	351.7 ± 172.1	6.07	<0.001
DHEA-S (mcg/dL)	3295 ± 125.1	167.4 ± 57.5	7.66	<0.001

(t-test for independent samples)

The two subgroups of the examined group classified as responders and nonresponders did not

significantly differ between themselves in terms of gender (men/women: 29/8 and 15/8 respectively; Pearson Chi-square =1.26, df = 1, p = 0.26), age (t = 0.34, p = 0.73), marital status (Pearson Chi-square = 1.41, df = 2, p = 0.49), education (Pearson Chi-square = 4.21, df = 3, p = 0.24), age of onset of the disorder (Z = 0.15, p = 0.88), duration of illness (Z = 0.32, p = 0.75), number of relapses (Z = 0.11, p = 0.9), number of hospital treatments (Z = 0.68, p = 0.49) and the type of antipsychotic agents - typical/atypical (Pearson Chi-square = 0.86, df =1, p = 0.35).

Graph 1 shows serum cortisol and DHEA-S levels in the subgroup of responders compared with the subgroup of nonresponders at baseline assessment point.

**Figure 1:**
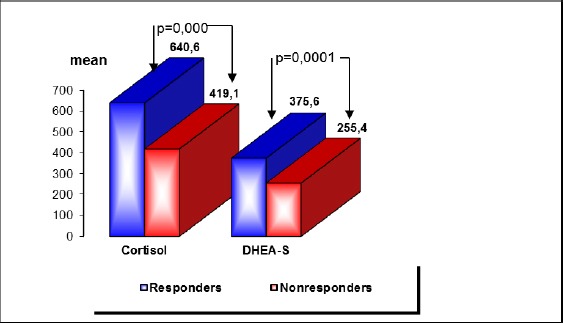
*Serum cortisol (nmol/L) and DHEA-S (mcg/dL) levels at baseline in responders and nonresponders*.

Table 2 shows mean serum cortisol and DHEA-S values after 3 and 6 weeks of antipsychotic therapy in responders and nonresponders.

**Table 2:** Serum cortisol and DHEA-S levels after 3 and 6 weeks of antipsychotic therapy in responders and nonresponders.

Hormone	Responders N=37	Nonresponders N=23
	**After 3 weeks**	
Cortisol (nmol/L)	530.9 ± 133.1	404.6 ± 119.2
DHEA-S (mcg/dL)	339.2 ± 132.9	239.9 ± 113.8
	**After 6 weeks**	
Cortisol (nmol/L)	449.9 ± 147.2	325.4 ± 125.1
DHEA-S (mcg/dL)	303.9 ± 121.0	224.9 ± 108.1

Across all three assessment points (baseline, after 3 and 6 weeks) the responders had a significantly higher serum cortisol and DHEA-S levels compared with nonresponders. Hormonal values significantly decrease during the study period of 6 weeks in both subgroups. Significant ‘group’ x ‘time’ interaction was found for hormone cortisol (p=0.002) (Table 3).

**Table 3:** Comparison of hormonal concentrations between responders and nonresponders across three points (MANOVA).

Serum stress hormones	t-value	Df		F	p
	**Responders vs non-responders**	
Hotelling-Lawley test	0.62	3	56	11.46	<0,001
Cortisol	1054548.030	1	58	28.683	<0001
DHEA-S	366603.017	1	58	10.747	0.002
	**Across three examination**	
Hotelling-Lawley test	0.862	6	226	16.24	<0.001
Cortisol	576305.421	2	116	44.38	<0.001
DHEA-S	77337.031	2	116	10.08	<0.001
	**Interaction**	
Hotelling-Lawley test	0.147	6	226	2.77	0.013
Cortisol	87261.088	2	116	6.72	0.002
DHEA-S	16020.123	2	116	2.09	0.13

## Discussion

Recently, studies on schizophrenia have increasingly focused on potential causative factors, such as structural and functional brain abnormalities. The assumption that alterations in cortisol and DHEA-S levels may have a role in changes in clinical presentation of several neuropsychiatric disorders, including schizophrenia, has been emphasized [2].

Our study showed that plasma cortisol levels were significantly elevated in the group of patients with schizophrenia compared with controls, which is in agreement with the results of most of the studies [1-10]. However there are studies reporting no significant differences between the schizophrenic patients and healthy controls in terms of cortisol levels [11, 28], as well as lower cortisol levels in patients with schizophrenia [12].

Examined serum DHEA-S levels in this study showed statistically significant higher levels in patients with schizophrenia compared to control subjects, which coincided with the results of most of the studies [2, 17-18]. In contrast, some other studies found decreased serum DHEA-S levels [20] and no different [11] in schizophrenia patients compared to healthy controls.

According to our results we can conclude that elevated serum cortisol and DHEA-S levels in patients with schizophrenia may play a role in the pathophysiology of schizophrenia and they may be used as a biological marker for the diagnosis of schizophrenia.

The present study also evaluated association between serum cortisol and DHEA-S levels and response to antipsychotic treatment in patients with schizophrenia. At baseline assessment point the subgroup of responders showed significantly higher serum cortisol and DHEA-S levels compared with the subgroup of nonresponders. Across all three assessment points the responders had a significantly higher serum cortisol and DHEA-S levels compared with nonresponders which is in agreement with the results of other study [21]. Our results suggest that elevated serum cortisol and DHEA-S levels may be related to positive response to antipsychotic treatment in patients with schizophrenia.

Higher serum cortisol and DHEA-S levels among responders compared with nonresponders may be explained by the differences in symptom severity on PANSS factors across three examination points. Our study showed that responders scored significantly higher on positive PANSS scale, delusions and suspiciousness compared with nonresponders which coincided with the results of other study [21]. Responders also showed greater reduction of the PANSS positive and negative scale scores across all three assessment points compared with nonresponders.

Future studies that examine serum cortisol and DHEA-S levels before and after the dexamethasone administration are needed to disentangle the baseline and feedback components of the HPA axis alteration in patients with schizophrenia.

## Conclusions

- Serum cortisol and DHEA-S levels were significantly elevated in the group of patients with schizophrenia compared with healthy controls.
- Elevated serum cortisol and DHEA-S levels may be used as a biological marker for the diagnosis of schizophrenia.
- The subgroup of responders had a significantly higher serum cortisol and DHEA-S levels compared with the subgroup of nonresponders.
- Elevated serum cortisol and DHEA-S levels may be related to positive response to antipsychotic treatment in patients with schizophrenia.
